# Changes in left ventricular function and coronary blood flow velocity during isocapnic hypoxia: A cardiac magnetic resonance imaging study

**DOI:** 10.1186/1532-429X-18-S1-P126

**Published:** 2016-01-27

**Authors:** Glen E Foster, Zixin Deng, Lindsey M Boulet, Puja K Mehta, Janet Wei, Zhaoyang Fan, Rohan Dharmakumar, C Noel, Bairey Merz, Debiao Li, Michael D Nelson

**Affiliations:** 1 grid.17091.3e0000000122889830Center for Heart, Lung, and Vascular Health, University of British Columbia, Kelowna, BC Canada; 2 grid.50956.3f0000000121529905Biomedical Imaging Research Institute, Cedars-Sinai Medical Center, Los Angeles, CA USA; 3 grid.50956.3f0000000121529905Heart Institute, Cedars-Sinai Medical Center, Los Angeles, CA USA

## Background

Cardiac stress testing is the standard of care for diagnosing ischemic heart disease. Traditional stress testing involves physical or pharmacological stress to induce hyperemia and/or increase myocardial oxygen demand. Physical stress is not possible in 100% of cases however, and pharmacological stress carries rare but serious risk. We asked whether acute isocapnic hypoxia could be utilized as an alternative cardiovascular stress test.

## Methods

Eight healthy male volunteers (31 + 4 yrs) were exposed to isocapnic hypoxia using a dynamic end-tidal forcing system. Left ventricular function and coronary artery blood flow velocity were measured by MRI (3T, Siemens). The end-tidal partial pressure of oxygen was maintained at 43 ± 0.3 mmHg, while the end-tidal partial pressure of carbon dioxide was controlled at baseline levels. Left ventricular ejection fraction was assessed using a free-breathing cine sequence (TE/TR = 1.08/46.74 ms; in-plane spatial resolution = 2.5 × 2.5 mm^2^; slice thickness = 8 mm). Coronary blood flow velocity was measured in the left anterior descending (LAD), left circumflex (LCX), and left main (LM) coronary arteries using a free-breathing, navigator-gated, Cartesian 2D phase-contrast (PC)-MRI sequence (temporal resolution = 26.4 ms; in-plane spatial resolution = 0.88 × 0.88 mm^2^; slice thickness = 7 mm; VENC = 40-80 cm/s in z-direction). Coronary cross-sectional area was assessed in 3 of 8 subjects (all in the left anterior descending coronary artery, LAD), using a 2D balanced steady-state free precession sequence (ECG-triggered and navigator gated; acquisition only in the quiescent phase; in-plane spatial resolution = 0.85 × 0.85 mm^2^; slice thickness = 7 mm).

## Results

During hypoxia arterial oxyhemoglobin saturation was reduced to 79 ± 1% and heart rate and systolic pressure increased by 47% and 4%, respectively (all P < 0.05). Hypoxia increased left ventricular ejection fraction from 66 + 1 to 74 + 1% (p< 0.01) and rate pressure product from 7057 + 639 to 10340 + 801 mmHg/beat/min (P < 0.01). Mean coronary flow velocity increased significantly in seven of the eight subjects (5 LAD, increasing from 17.9 + 2.1 to 25.6 + 1.2 cm/s; 1 LCX, increasing from 20.1 to 38.8 cm/s; and 1 LM, increasing from 18.6 to 40.0 cm/s). Poor image quality prevented analysis of coronary flow velocity in 1 subject. The change in coronary flow velocity was proportional to the change in myocardial oxygen demand (P = 0.26). Coronary cross-sectional area was measured in three subjects and found to remain constant (22.3 + 4.5 vs. 22.4 + 5.3 mm^2^, p = ns, baseline vs. hypoxia, respectively).

## Conclusions

This is the first MRI study to simultaneously evaluate cardiac function and coronary blood flow in response to acute isocapnic hypoxia using dynamic end-tidal forcing. The results support the use of hypoxia as a unique cardiovascular stress test. Further investigation is required to determine the feasibility and efficacy of its use in targeted patient populations.

